# A Fifteen‐Gene Classifier to Predict Neoadjuvant Chemotherapy Responses in Patients with Stage IB to IIB Squamous Cervical Cancer

**DOI:** 10.1002/advs.202001978

**Published:** 2021-03-18

**Authors:** Xun Tian, Xin Wang, Zifeng Cui, Jia Liu, Xiaoyuan Huang, Caixia Shi, Min Zhang, Ting Liu, Xiaofang Du, Rui Li, Lei Huang, Danni Gong, Rui Tian, Chen Cao, Ping Jin, Zhen Zeng, Guangxin Pan, Meng Xia, Hongfeng Zhang, Bo Luo, Yonghui Xie, Xiaoming Li, Tianye Li, Jun Wu, Qinghua Zhang, Gang Chen, Zheng Hu

**Affiliations:** ^1^ Department of Obstetrics and Gynecology Tongji Hospital Tongji Medical College Huazhong University of Science and Technology Jiefang Avenue 1095# Wuhan Hubei 430030 China; ^2^ Department of Obstetrics and Gynecology Academician expert workstation, The Central Hospital of Wuhan Tongji Medical College Huazhong University of Science and Technology Jiefang Avenue 1095# Wuhan Hubei 430030 China; ^3^ Department of Gynecological and Oncology The First Affiliated Hospital of Sun Yat‐sen University Zhongshan 2nd Road, Yuexiu Guangzhou Guangdong 510080 China; ^4^ Department of Gynecological and Oncology Hunan Cancer Hospital The Affiliated Cancer Hospital of Xiangya School of Medicine Central South University Jiefang Avenue 1095# Wuhan Hubei 430030 China; ^5^ NGS Research Center Novogene Co, Ltd Building 301, Zone A10 Jiuxianqiao Beijing 100015 China; ^6^ Department of Pathology The Central Hospital of Wuhan Tongji Medical College Huazhong University of Science and Technology Shengli Street 26#, Jiang'an District Wuhan Hubei 430030 China

**Keywords:** CRISPR/Cas9‐based library screening, neoadjuvant chemotherapy, precision medicine, whole exon sequencing

## Abstract

Neoadjuvant chemotherapy (NACT) remains an attractive alternative for controlling locally advanced cervical cancer. However, approximately 15–34% of women do not respond to induction therapy. To develop a risk stratification tool, 56 patients with stage IB‐IIB cervical cancer are included in 2 research centers from the discovery cohort. Patient‐specific somatic mutations led to NACT non‐responsiveness are identified by whole‐exome sequencing. Next, CRISPR/Cas9‐based library screenings are performed based on these genes to confirm their biological contribution to drug resistance. A 15‐gene classifier is developed by generalized linear regression analysis combined with the logistic regression model. In an independent validation cohort of 102 patients, the classifier showed good predictive ability with an area under the curve of 0.80 (95% confidence interval (CI), 0.69–0.91). Furthermore, the 15‐gene classifier is significantly associated with patient responsiveness to NACT in both univariate (odds ratio, 10.8; 95% CI, 3.55–32.86; *p* = 2.8 × 10^−5^) and multivariate analysis (odds ratio, 17.34; 95% CI, 4.04–74.40; *p* = 1.23 × 10^−4^) in the validation set. In conclusion, the 15‐gene classifier can accurately predict the clinical response to NACT before treatment, representing a promising approach for guiding the selection of appropriate treatment strategies for locally advanced cervical cancer.

## Graphical abstract

In a multicenter study of 158 patients with stage IB‐IIB cervical cancer, a 15‐core‐gene classifier is developed by integrated analysis of whole‐exome sequencing and CRISPR library screening. By predicting cervical cancer patients who would benefit from neoadjuvant chemotherapy, the classifier has the potential to guide the proper selection of treatment modalities, implicating a significant advance toward individualizing therapy.

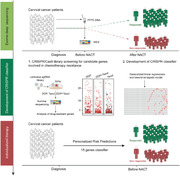

## 1 Introduction

Cervical cancer remains the most common cancer in women globally, with approximately 570 000 newly diagnosed patients and 311 000 deaths each year.^[^ ^1^ ^]^ Patients with stage IB to IIB cervical carcinoma can be treated by concurrent chemoradiation (CCRT) or radical hysterectomy (RH) including pelvic lymph node dissection.^[^ ^2^ ^]^ CCRT is preferred in most developed countries.^[^ ^3^ ^]^ However, pelvic radiation may lead to side effects including ovarian failure in premenopausal women,^[^ ^4^ ^]^ radiation cystitis,^[^ ^5^ ^]^ rectal bleeding,^[^ ^6^ ^]^ and vaginal stenosis.^[^ ^6^, ^7^ ^]^ Another treatment recommendation is RH with pelvic lymph node dissection.^[^ ^8^, ^9^ ^]^ However, in addition to the fact that large tumors are difficult for surgeons to resect, the large proportion of vascular carcinoma emboli found in locally advanced tumor patients means there is greater potential for it to spread throughout the body.^[^ ^10^ ^]^ It is, therefore, necessary to explore more effective treatment methods for stage IB to IIB cervical cancer.

Neoadjuvant chemotherapy (NACT) followed by RH is considered an attractive strategy for patients with stage IB to IIB cervical cancer.^[^ ^9^, ^11^ ^]^ Cervical cancer has a high responsiveness rate to taxane‐ and platinum‐based chemotherapy.^[^ ^12^ ^]^ The potential advantages of NACT include: i) reduction of tumor size and eradication of micro‐metastasis,^[^ ^13^ ^]^ providing better local control for the following RH; ii) a better toxicity profile compared to CCRT, without compromising survival benefits.^[^ ^14^ ^]^ Although whether NACT should be the better treatment for locally advanced cervical cancer (LACC) remained debatable, it has the potential to become a standard of care, even in regions where radiation therapy is available. Today, NACT has been largely used in England,^[^ ^15^ ^]^ German,^[^ ^16^, ^17^ ^]^ Italy,^[^ ^18^ ^]^ Bulgaria,^[^ ^19^ ^]^ Belgium,^[^ ^20^ ^]^ China,^[^ ^21^ ^]^ South Korea,^[^ ^22^ ^]^ Mexico,^[^ ^23^ ^]^ and Japan.^[^ ^24^ ^]^ Different experiences from Europe, Asia, and South America have shown an improved benefit at 5‐year survival when using NACT followed by RH or chemoradiation in the LACC cases.^[^ ^25^, ^26^ ^]^

Despite the advantages, one major concern of NACT is that approximately 15–34% of patients do not respond to induction therapy, thus unnecessarily delaying effective local therapy.^[^ ^13^, ^27^ ^]^ These findings highlight the need for establishing selection criteria for patients who would benefit most from NACT. With the advancement of high‐throughput next‐generation sequencing, comprehensive genomic profiles have been obtained and systematically analyzed to characterize tumor‐associated mutations that can predict treatment response.^[^ ^28^ ^]^ This holds promise for predicting the benefits of chemotherapy in breast cancer,^[^ ^29^ ^]^ gastric cancer,^[^ ^30^ ^]^ and chronic myelomonocytic leukemia.^[^ ^31^ ^]^

In this study, we report the development and validation of a multigene panel for predicting the clinical response to NACT in patients with stage IB to IIB cervical cancer. The prediction model allows the selection of patients who would benefit most from NACT, while sparing other patients toxic side effects and delays in CCRT or RH. This provides a more careful and personalized risk assessment beyond current clinical parameters.

## 2 Results

### 2.1 Clinical Cohort

**Figure 1:**
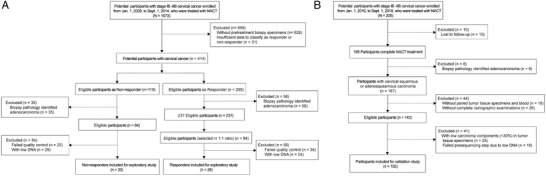
Flow diagram demonstrating the breakdown of study population. A) Discovery cohort; B) Validation cohort.

In the discovery nested case‐control study, 56 patients were identified from 1073 cervical cancer patients (stage IB to IIB) who underwent NACT in our Clinical Database and Biobank, based on a 1:1 (non‐responder: responder) ratio^[^ ^32^ ^]^ (**Figure** **1**A; Table S1, Supporting Information). The clinical response to NACT was determined by measuring the dynamic changes in tumor area (multiplication of longest diameter by the greatest perpendicular diameter) during each treatment cycle. The clinical response criteria of the World Health Organization (WHO) were used^[^ ^33^ ^]^ as follows: complete tumor disappearance was defined as complete response (CR); tumor size decreasing by more than 50% was defined as partial response (PR); tumor size decreasing by less than 50% was defined as stable disease (SD); and the emergence of new lesions or an increase in tumor size was defined as progressive disease (PD). CR or PR patients were referred to as responders, whereas SD or PD patients were referred to as non‐responders. In the training cohort, 26 of 56 (46.43%) patients demonstrated responsiveness to NACT, including one (1.79%) with CR and 25 (44.64%) with PR. 30 of 56 (53.57%) patients demonstrated non‐responsiveness to NACT, including 29 (51.79%) with SD and one (1.79%) with PD (Table S2, Supporting Information).

In the independent validation cohort, 205 participants were enrolled between 1st January 2016 and 1st September 2018. Of these patients, 103 were excluded for biopsy pathology results, insufficient samples, and unqualified DNA (Figure 1B). The baseline characteristics are reported in Table S3, Supporting Information. In this study, to compare the WHO and RECIST criteria, vaginal ultrasound data of 14 patients in the validation cohort were randomly verified by magnetic resonance imaging (MRI) based on RECIST criteria. The results showed WHO criteria were in good concordance with RECIST criteria (Figure S1, Supporting Information). Clinical responses were observed in 80 of 102 (78.43%) patients, including 34 (33.33%) with CR and 46 (45.10%) with PR. Clinical non‐responses were seen in 22 of 102 (21.57%) patients, including 18 (17.65%) with SD and 4 (3.92%) with PD (Table S2, Supporting Information).

### 2.2 Copy Number Variation Does Not Correlate With NACT Response in Cervical Cancer Patients with Stage IB to IIB

Previous reports have revealed that copy number variation (CNV) and somatic mutations are prevalent in cervical cancer,^[^ ^34^, ^35^ ^]^ and are potentially associated with chemotherapy resistance.^[^ ^36^, ^37^ ^]^ To identify a predictive genomic signature via exploratory research, we first applied an Affymetrix OncoScan microarray to pairwise tumor‐normal samples from 47 patients in the training set. This was done to detect potential genome‐wide CNVs for predicting response to NACT. GISTIC2.0 analysis (with a threshold of *q* < 0.25) revealed 14 focal amplifications and 28 focal deletions (Figure S2, Table S4 in data file S1, Supporting Information). Among these 42 focal CNVs, we confirmed recurrently amplified regions at 3q28 (TP63, 81%, *q* = 0.002) and deleted regions at 2q37.1 (CCL20, ABCB6, 47%, *q* = 1.33 × 10^−7^) and 17q25.3 (FOXK2, 21%, *q* = 3.05×10^−5^), consistent with previous studies of cervical cancer.^[^ ^34^ ^]^ In addition to these findings, we also identified multiple novel recurrent focal amplification events, including the most significant regions 11q22.2 (YAP1, BIRC2, and BIRC3, 45%, q = 1.36 × 10^−5^), 22q11.23 (GSTT1, GSTTP1, and GSTTP2, 19%, *q* = 0.004), and 18p11.31 (EPB41L3, 17%, *q* = 0.05). We also observed multiple deleted regions including 11q23.3 (CHEK1, ATM, 62%, *q* = 2.79 × 10^−5^), 19p13.3 (STK11, 36%, *q* = 7.59 × 10^−5^), and 13q13.1 (BRCA2, 38%, *q* = 5 × 10^−4^). These participate in the homologous recombination pathway, as previously reported.^[^ ^38^ ^]^

We first compared the gain/loss status of previously reported genes to platinum/taxol resistance,^[^ ^39^ ^]^ including CCNE1, BRCA2, ERCC1, and TEKT4. When investigating these genes, we found no significant differences between therapy responders and non‐responders (*p* > 0.05, Table S5 in data file S1, Supporting Information). We then investigated whether the gain/loss status of driver genes may play a role in the response and resistance to NACT. Hierarchical clustering of variable driver genes did not produce two clear segregated groups (*p* > 0.05, Table S5 in data file S1, Supporting Information).

**Figure 2:**
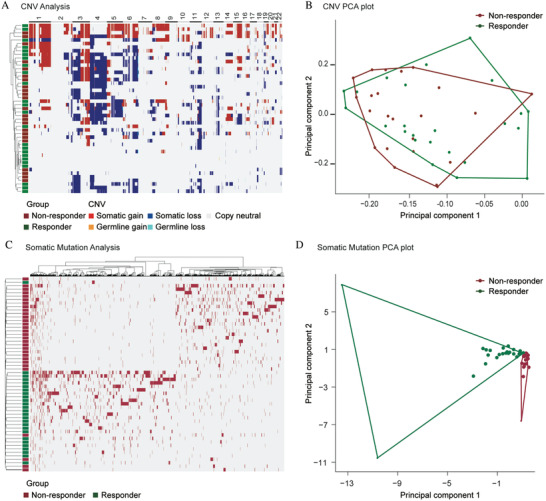
CNV and somatic mutation analysis in the training cohort. A) Heatmaps of unsupervised hierarchical clustering of copy number variation. Bar colors indicate clinical response to NACT: red indicates a non‐responder, and green indicates a responder. Colored rectangles on heatmaps show CNVs and single nucleotide variations (SNVs)/Indels. B) PCA of CNVs. C) Heatmaps of unsupervised hierarchical clustering of somatic mutations. D) PCA of somatic mutations.

Next, we investigated any significant predicted signatures based on the total number of genes with copy number gain/loss per sample. However, hierarchical clustering of the training cohort based on the aberrant somatic CNV profile failed to achieve perfect segregation of NACT‐sensitive and resistant patients (**Figure** **2**A). We further performed principal component analysis (PCA) on the data of aberrant somatic CNVs from each patient. As seen in Figure 2B, non‐responders and responders showed similar intra‐group diversity in genomic instability, with indistinguishable PCA values. These did not have distinct CNV profiles.

### 2.3 Specific Somatic Mutation Profiles of NACT‐Sensitive and Resistant Cervical Cancer Patients with Stage IB to IIB

Given the lack of clear differences in copy number gain/loss status between NACT responders and non‐responders, we speculated that there may be differences in key somatic gene mutations between NACT‐sensitive and resistant patients at diagnosis. Building these mutational profiles could predict drug responses in cervical cancer patients.^[^ ^40^ ^]^ For this purpose, we further conducted whole exon sequencing (WES, > 200×) on pairwise tumor‐normal samples using the same 47 cases and an additional 9 cases. In the dataset, a total of 16 928 somatic non‐silent mutations were discovered, including 16 015 point mutations and 913 indels. A summary of somatic alterations is shown in Table S4 in data file S1, Supporting Information. In addition to the previously reported genes PIK3CA (28.57%), EP300 (16.07%), CASP8 (10.71%), and STK11 (7.14%), we also identified novel recurrent mutations in CLPS (16.07%), ICAM4 (8.93%), OSTC (8.93%), ST6 Beta‐Galactoside Alpha‐2,6‐Sialyltransferase 2 (ST6GAL2) (7.14%), WIPF2 (7.14%), and POM121 (5.36%).

To study whether these mutated genes could lead to different therapy outcomes, we performed hierarchical clustering of the training cohort. In this analysis, we focused on the obvious difference in mutation genes between responders and non‐responders. We identified 751genes in the non‐responder group (Mut_insen–Mut_sen ≥ 2) and 909 genes in the responder group (Mut_sen–Mut_insen ≥ 2). Hierarchical clustering of the samples using these genes was sufficient to achieve good segregation of NACT‐sensitive and resistant patients (Figure 2C; Table S6 in data file S1, Supporting Information). We next performed PCA on the somatic mutation gene data (Figure 2D). As expected, non‐responders and responders could be clearly divided into two different clusters. These findings indicated that differences in somatic mutations may correlate with the likelihood of a patient responding to NACT treatment.

**Figure 3:**
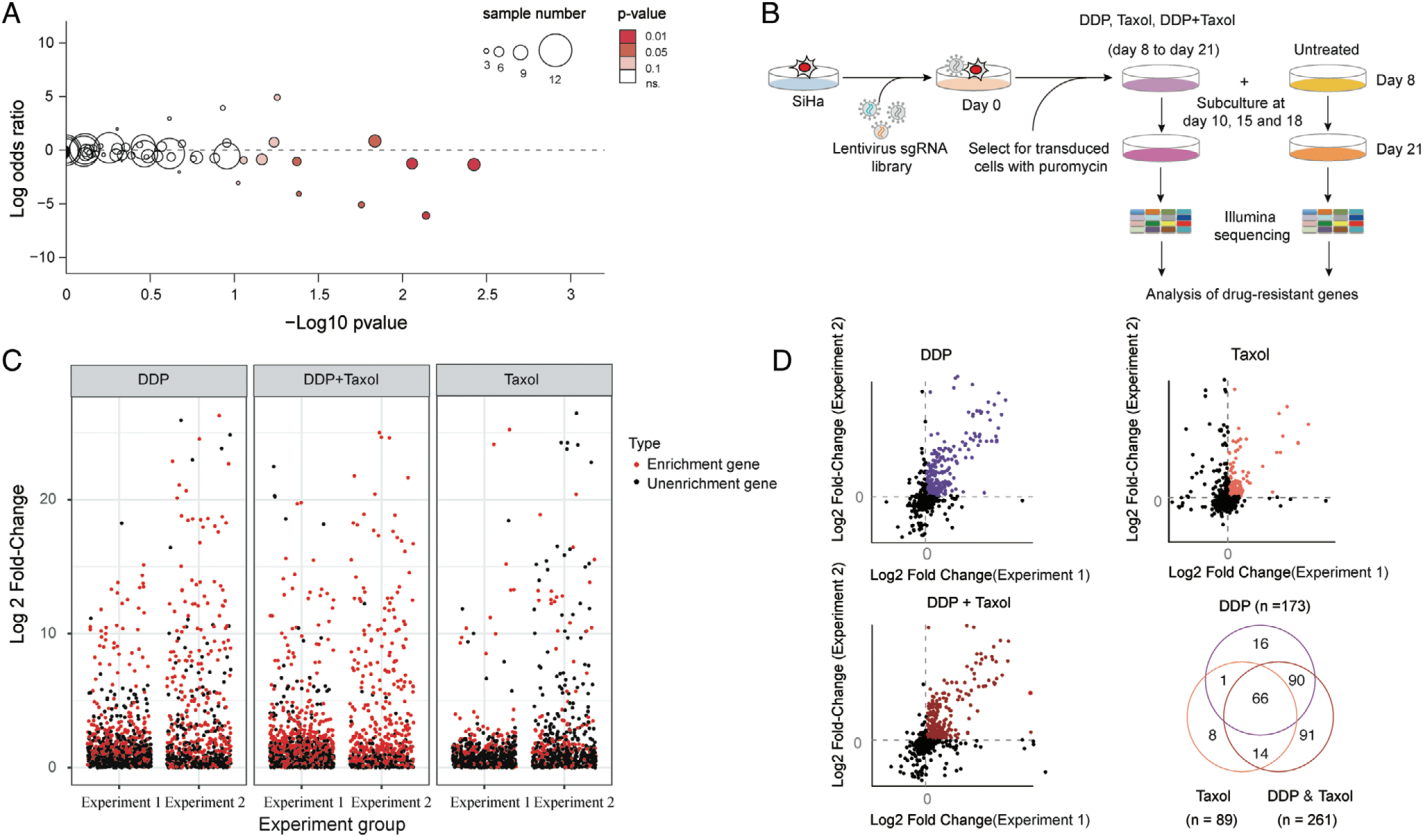
Genetic mutations associated with drug resistance. A) Volcano plot representation of odds ratio analysis showing the risk of drug resistance (log 2 odds ratio) and significance (*p*‐value, Fisher's exact test) of associations between somatic mutations and NACT response. Each circle represents a gene, the size is proportional to the sample number mutated in the cohort, and the color indicates the *p*‐value. B) Schematic outline of the screening pipeline. C) Scatter plots showing enrichment of specific genes in the SiHa cell line after drug treatment. Duplicate experiments were arranged for each toxin screening, each with three replicates. The enrichment level was calculated as log 2 (Exp:Ctrl) of the normalized read counts. The enrichment genes in duplicate experiments were marked in red, and un enrichment genes were marked as dark. D) Filtration of three drug‐resistant experiments of CRISPR/Cas9 screens. Dot plots show the selection of consistently changed genes in two independent technical experiments. Venn diagram shows the final selected gene set.

We next performed enrichment analysis to recognize genes that were selectively mutated in the non‐responders (Mut_insen–Mut_sen ≥ 2). After filtration, only 744 candidate genes were retained (*p* < 0.25) (**Figure** **3**A; Table S7 in data file S1, Supporting Information). In gene enrichment analysis, 722 of 17 913 tested Gene Ontology (GO) categories were significantly enriched in the 744‐candidate gene after multiple testing corrections via the Benjamini–Hochberg (BH) method (Figure S3, Supporting Information). We found strong enrichment for biological processes related to histone modification (*p* = 1.36 × 10^−6^), covalent chromatin modification (*p* = 2.75 × 10^−6^), regulation of small GTPase‐mediated signal transduction (*p* = 3.86 × 10^−6^), and regulation of Ras protein signal transduction (*p* = 1.04 × 10^−5^) (Table S8 in data file S1, Supporting Information). This suggests that these mutations likely contribute to chemoresistance via inducing dysfunction of the aforementioned pathways.

### 2.4 CRISPR/Cas9 Library Screening for Candidate Genes Involved in Chemotherapy Resistance

To gain insight into the functions by which mutations could influence the response to cisplatin/taxol, we constructed a custom‐designed high‐throughput CRISPR/Cas9 library based on 744 genes. This had an average coverage of 3–6 sgRNAs per gene. We then performed loss‐of‐function screening in human cervical cancer cell line SiHa (Figure 3B). Replicate screens with cisplatin/taxol narrowed potential candidates to a total of 286 genes related to drug resistance (Figure 3C; Table S9 in data file S1, Supporting Information). We determined 173, 261, and 89 inactivated genes that caused resistance to cisplatin, cisplatin plus taxol, and taxol in the SiHa cell line, respectively (fold changes > 1 and *p* values < 0.1) (Figure 3D). Our highest ranking genes included several genes previously implicated in platinum resistance, such as ARID1A (*p* = 1.86 × 10^−36^),^[^ ^41^ ^]^ and multiple genes impacting double‐strand break repair or regulating responses to DNA damage stimulus including E3 ligase UBR5 (*p* = 8.9 × 10^−28^)^[^ ^42^ ^]^ and POLQ (*p* = 7.1 × 10^−4^).^[^ ^43^ ^]^ Additionally, several other druggable genes that play a key role in multidrug resistance were identified. These included ABCB1 (*p* = 2.35 × 10^−14^),^[^ ^44^ ^]^ CTC1 (*p* = 1.38 × 10^−5^),^[^ ^45^ ^]^ and SLAM family receptor LY9 (*p* = 4.71 × 10^−9^).^[^ ^46^ ^]^ (Table S9 in data file S1, Supporting Information). These were among the most downregulated genes discovered via in vitro screening. Our results suggested the above mutations associated with the resistance of cisplatin/taxol.

### 2.5 Development and Comparison of Different Genomic Classifiers

**Figure 4:**
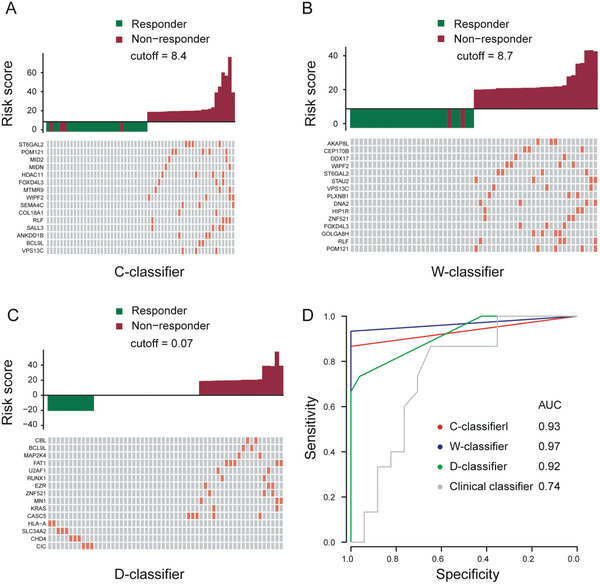
Risk score calculated by three classifier and Receiver Characteristic Operator (ROC) curves. A–C) Upper‐left panel: risk score distribution of the C‐classifier, W‐classifier, and D‐classifier and the response status of 56 patients in the training cohort. Lower‐left panel: the mutational status of the C‐classifier, W‐classifier, and D‐classifier in the 56 patients. D) ROC curves of the C‐classifier, W‐classifier, D‐classifier, and clinical classifier in the training cohort.

In CRISPR screening experiments, 286 candidate genes were enriched according to their functional contributions to chemotherapy resistance. Given that our goal was to identify a candidate model for dissecting cervical cancer patients with stage IB to IIB who would benefit most from NACT, we used generalized linear regressions to select variables and construct a logistic prediction model. This allowed us to develop a CRISPR‐classifier (C‐classifier) consisting of 15 core genes (Table S10, Supporting Information) in the training cohort. A risk score was calculated for each patient using a formula derived from the mutational status of these 15 genes weighted by their regression coefficient (Method). The risk scores of the patients in the training set ranged from 1.3 to 21.8. We set a cut‐off (8.4) using the maximum Youden index strategy of receiver operating characteristic (ROC) curves for differentiating non‐responders from responders. Patients with a threshold below this cut‐off were considered to be a responder to NACT, whereas patients performing above this cut‐off were considered non‐responders (**Figure** **4**A).

Then, using the same formula, the original 744 genes identified by WES were applied to develop a second model termed the WES‐classifier (W‐classifier) (Figure 4B). In an attempt to refine the model, the 744 genes were then narrowed down to cancer driver genes from the Cancer Gene Census (CGC)^[^ ^47^ ^]^ to develop a Driver‐classifier (D‐classifier) (Figure 4C). In comparison with these genetic models, we also developed a clinical classifier base on clinical variables, including age, pathological differentiation, clinical stage, and SCC‐pretreatment.

Next, we used ROC curves analysis to evaluate the clinical validity of the three predictive classifiers and the clinical classifier for discriminating the cervical cancer patients with stage IB to IIB not responding to NACT. In the training set, the C‐classifier, integrated with information regarding gene functions, yielded an area under the curve (AUC) of 0.93 (95% confidence interval (CI), 0.87–1.00). This was similar to the W‐classifier (AUC, 0.97; 95% CI, 0.92–1.00) and the D‐classifier (AUC, 0.92; 95% CI, 0.86–0.97), but was greater than that of the clinical‐classifier (AUC, 0.74; 95% CI, 0.56–0.92) (Figure 4D). Although these three genetic classifiers demonstrated good clinical performance in the discovery set, the predicted reproducibility and validity of the three models should be tested in an external cohort.

### 2.6 Primary Resistance to Cisplatin/Paclitaxel is Associated with ST6GAL2 Downregulation

**Figure 5:**
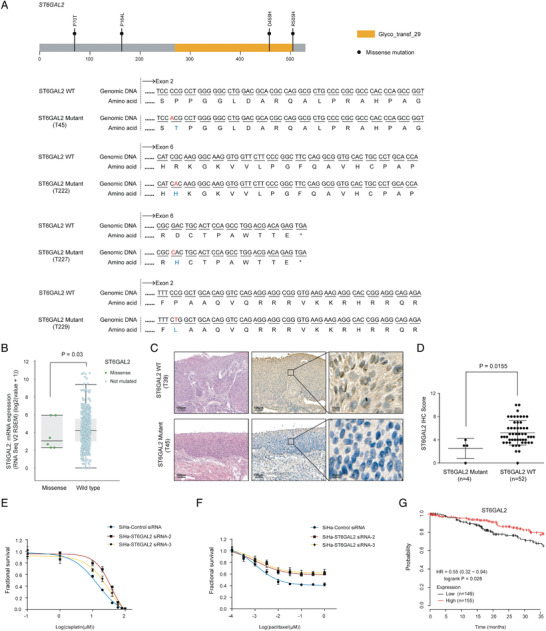
ST6GAL2 loss leads to cisplatin/paclitaxel resistance. A) gDNA and amino acid sequences of ST6GAL2^mut^, respectively. B) ST6GAL2 mRNA level in the samples with missense and wild type. C,D) ST6GAL2 expression in mutation and wild genotype pretreatment tumor biopsies. E,F) Viability of SiHa cells with siRNA directed at ST6GAL2 after treatment with cisplatin/paclitaxel in vitro. G) Kaplan–Meier analysis of OS in a cervical squamous cell carcinoma dataset (n = 304).

To further validate the influence of model genes on drug resistance, we selected ST6GAL2 to confirm the biological functions of its deleterious mutations. This was based on its highest coefficient in the C‐model. For WES analysis, we identified four missense mutations in ST6GAL2 (**Figure** **5**A, Table S4, Supporting Information) in the training group. These mutations involved missense events at amino acid positions 505, 458, 164, and 70. ST6GAL2 encoded a protein of 529 amino acids and contained the Glyco_trans_29 domain. Without exception, the ST6GAL2 mutant group exhibited resistance to NACT. In view of the strong association between ST6GAL2 deleterious mutations and drug resistance, we first verified whether gene mutations affect gene expression in TCGA datasets, (https://www.cbioportal.org/). In the datasets, 475 samples with ST6GAL2 wild type and 6 samples with ST6GAL2 missense mutation were included for analysis. As shown in Figure 5B, the ST6GAL2 mRNA level of the samples with ST6GAL2 missense mutation was lower than that of the samples with wild type ST6GAL2 (*p* = 0.03). We further sought to examine the impact of ST6GAL2 mutations on protein expression levels in tumor tissue. We performed immunohistochemical (IHC) staining of ST6GAL2 in paired mutation and wild genotype pretreatment tumor biopsies from the discovery sample. ST6GAL2 was localized in the cytoplasm of tumor tissue (Figure 5C). In the ST6GAL2^mut^ cases, lower ST6GAL2 protein expression was observed in tumor tissue and revealed resistance to NACT (*p* = 0.0155) (Figure 5D).

To further functionally validate the association of ST6GAL2 deleterious mutations with drug resistance, we performed a siRNA knockdown of ST6GAL2. We found that this knockdown conferred resistance to paclitaxel/cisplatin in cell survival assays (Figure 5E,F). Additionally, by applying the ST6GAL2 gene to 304 patients in a cervical squamous cell carcinoma dataset (http://kmplot.com/analysis/), we found that poor prognosis was associated with low ST6GAL2 expression (*p* = 0.028) (Figure 5G).

### 2.7 Other Model Gene Mutations Abrogated the Effect of Cisplatin/Paclitaxel

**Figure 6:**
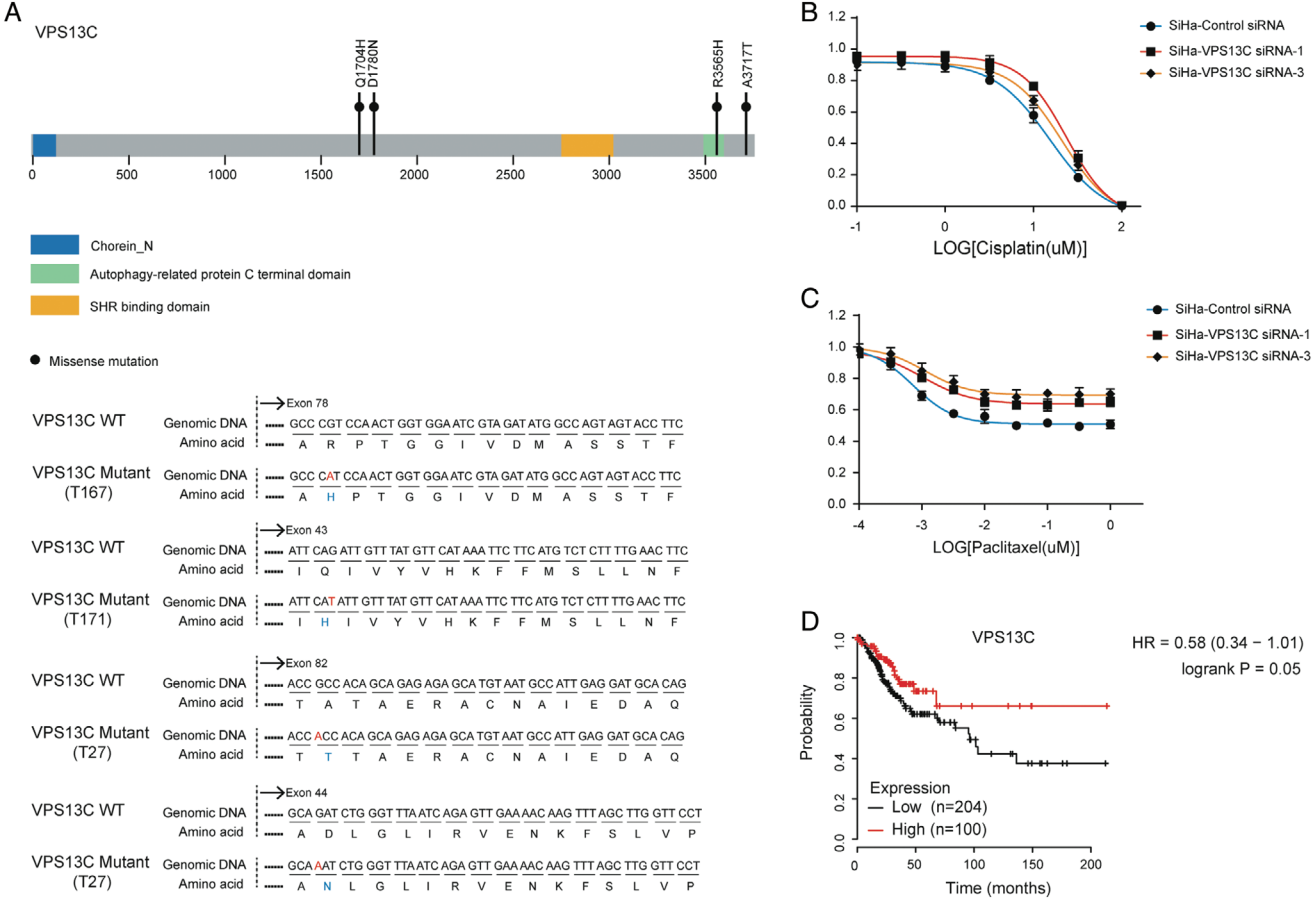
VPS13C gene loss leads to cisplatin/paclitaxel resistance. A) gDNA and amino acid sequences of VPS13C^mut^, respectively. B,C) Viability of SiHa cells with siRNA directed at VPS13C after treatment with cisplatin/paclitaxel in vitro. D) Kaplan–Meier analysis of OS in a cervical squamous cell carcinoma dataset (n = 304).

We then focused our investigation on VPS13C, which had the minimum coefficients in our C‐model. In the training group, four missense mutations were identified in 3 samples (**Figure** **6**A, Table S6, Supporting Information). To determine whether these deleterious mutations led to increased resistance to cisplatin/paclitaxel, we further performed a siRNA knockdown of VPS13C. Following the knockdown of the gene with the two most effective siRNAs, we found that VPS13C mutations induced resistance to both cisplatin and paclitaxel treatment in SiHa cells (Figure 6B,C). Analysis of the Kaplan–Meier plot of the cervical squamous cell carcinoma dataset showed that low VPS13C expression was associated with a reduction in overall survival (OS) (*p* = 0.05) (Figure 6D).

Then, we tested the functions of the other genes in the CRISPR model in SiHa cells, including POM121, FOXD4L3, MID2, etc. Following the knockdown of these genes with the two most effective siRNAs of each gene, we found that candidate gene mutations induced resistance to cisplatin or/and paclitaxel treatment in SiHa cells (Figure S4, Supporting Information).

### 2.8 Validation in an Independent Clinical Cohort

**Figure 7:**
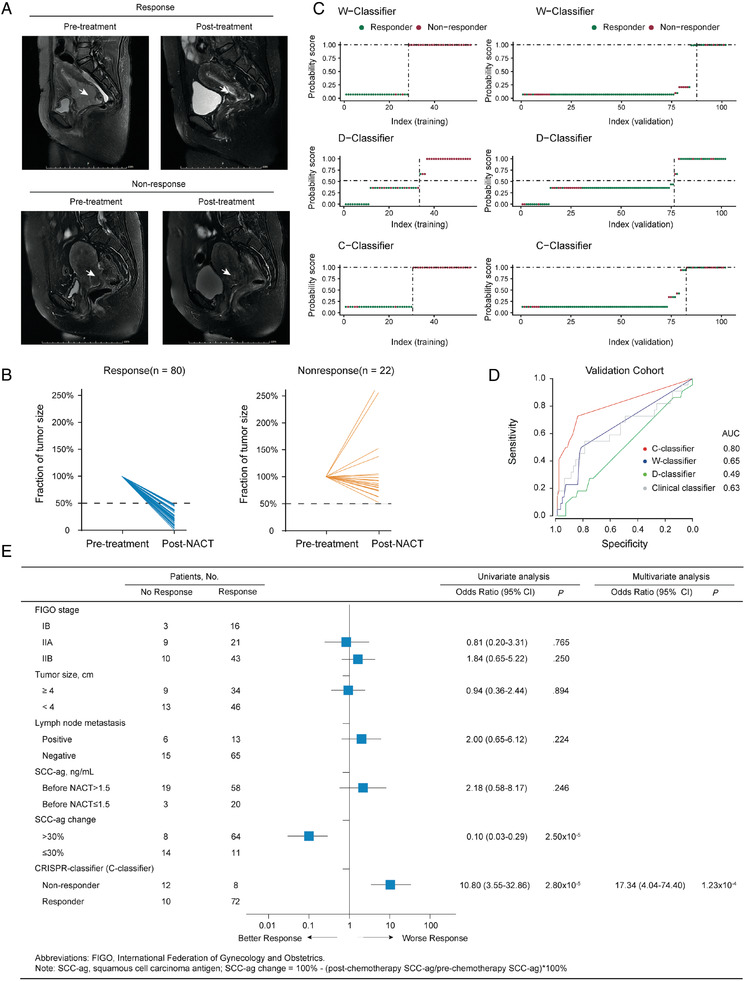
Performance of three genomic classifiers and ability to discriminate between responder and non‐responder patients in an independent validation cohort. A) Sagittal T2‐weighted MRI of the pelvis showing the cervical tumor. B) Relative change in cervical cancer, measured by 3D ultrasound in the validation cohort. C) Probability plot based on the C‐classifier, W‐classifier, and D‐classifier for correct class prediction. Training and Validation set: red circles indicate non‐responder; green circles indicate responder. D) ROC curves of the C‐classifier, W‐classifier, D‐classifier, and clinical classifier in the validation cohort. E) Association of Clinical Variables, C‐classifier With Response to NACT.

To explore the value of our classifiers in predicting NACT outcome, 102 diagnostic cervical cancer specimens from patients enrolled in an external prospective cohort from NCT03229187 were collected and subjected to WES. All of these patients had been treated with the same course of NACT. The genomic classifiers that had been developed using the training cohort were applied blindly to these samples, without any prior knowledge of the therapeutic response labels for this cohort. The clinical response to NACT was determined by measuring the dynamic changes in tumor size during each cycle of treatment (**Figure** **7A,B**).

The ability to predict whether a patient (stage IB to IIB) will not respond to NACT has important clinical implications. To evaluate the potential value of our predicted models in clinical practice, we analyzed the distribution of the probability scores across discovery and validation studies for the C‐classifier, W‐classifier, and D‐classifier. Probability scores, ranging from 0 to 1, were calculated for the prediction of both non‐responders and responders. As shown in Figure 7C, the probability of being a non‐responder was plotted with high values pertaining to non‐responders and low values corresponding to responders.

For the C‐classifier, 92.86% of samples in the training set were classified correctly. When applied to the validation set, C‐classifier showed a good discriminative capacity for the prediction of non‐responders from responders with a specificity of 90% and the AUC of 0.80 (95% CI, 0.69–0.91) (**Table** **1**, Figure S6, Supporting Information). In contrast, the AUC of W‐classifier and D‐classifier significantly decreased in the validation set [0.65 (95% CI, 0.53–0.76) for W‐classifier, and 0.49 (95% CI, 0.37–0.62) for D‐classifier] (Figure 7D). Compared to W‐classifier and D‐classifier, the C‐classifier demonstrated better performance in the independent validation cohort because it integrated the biological data of CRISPR library screening to narrow down the functional biomarker. The C‐classifier was further tested by down‐sampling the validation data to examine the model stability. As Figure S5, Supporting Information, showed, the AUC slightly changed around the AUC (0.8) of total validation data (Figure S5A,C,E, Supporting Information) and the accuracy slightly floated around the accuracy (82.35%) of total validation data (Figure S5B,D,F, Supporting Information). Overall, when combined with information regarding gene functions, the classifier based on the somatic mutation profile of patients (stage IB to IIB) demonstrated excellent predictive ability for determining patients who were not responding to NACT.

**Table 1:** Prediction of model performance in the discovery and validation cohorts

	Patients, No.	Accuracy, [%]	Sensitivity, [%] (SE)	Specificity, [%] (SE)	PPV, [%] (SE)	NPV, [%] (SE)	AUC (95% CI)	*P*
Training Cohort W‐classifier	56	96.43	93.33 (4.56)	100 (0)	100 (0)	92.86 (4.87)	0.97 (0.92–1.00)	8.62E–11
Validation Cohort W‐classifier	102	73.53	22.73 (8.93)	87.50 (3.70)	33.33 (12.17)	80.46 (4.25)	0.65 (0.53–0.76)	3.26E–03
Training Cohort D‐classifier	56	83.93	73.33 (8.07)	96.15 (3.77)	95.65 (4.25)	75.76 (7.46)	0.92 (0.86–0.97)	1.87E–08
Validation Cohort D‐classifier	102	64.71	27.27 (9.49)	75.00 (4.84)	23.08 (8.26)	78.95 (4.68)	0.49 (0.37–0.62)	5.47E–01
Training Cohort C‐classifier	56	92.86	86.67 (6.21)	100 (0)	100 (0)	86.67 (6.21)	0.93 (0.87–1.00)	8.32E–10
Validation Cohort C‐classifier	102	82.35	54.55 (10.62)	90.00 (3.35)	60.00 (10.95)	87.80 (3.61)	0.80 (0.69–0.91)	3.56E–08

Abbreviations: Positive predictive value, PPV; Negative predictive value, NPV.

The associations between the response to NACT and the C‐classifier, FIGO stage, tumor size, lymph node metastasis, and serum SCC‐ag levels were tested using logistic regression analysis (Figure 7E). The C‐classifier was significantly associated with chemotherapy response in terms of changes in tumor volumes in univariate analysis (odds ratio, 10.80; 95% CI, 3.55–32.86; *p* = 2.8 × 10^−5^). The classifier remained an independent factor for predicting clinical response to NACT when adjusting for clinical factors in multivariate analysis (odds ratio, 17.34; 95% CI, 4.04–74.40; *p* = 1.23 × 10^−4^). Interestingly, the decreased serum SCC‐ag levels (> 30%) demonstrated a predictive effect for clinical response to NACT (odds ratio, 0.10; 95% CI, 0.03–0.29; *p* = 2.50 × 10^−5^) in univariate analysis, but could not be applied at the time of diagnosis. The other clinical parameters did not affect the response to NACT in the validation cohort (*p* ≥ 0.05).

## 3 Discussion

Previous reports have revealed that CNVs and somatic mutations are prevalent in cervical cancer,^[^ ^34^, ^35^ ^]^ and are potentially associated with chemotherapy resistance.^[^ ^37^ ^]^ In this study, both genome‐wide CNV analysis and WES were conducted to study the genomic characteristics associated with clinical response to NACT. Interestingly, we discovered that whole genome CNVs did not correlate with therapeutic sensitivity or resistance to NACT. Instead, distinctive DNA mutational profiles were identified for chemotherapy responders and non‐responders (Figure 2C). We analyzed the mutational status of these candidate genes in the validation samples. As expected, 552 of 744 (74.2%) mutated genes had been verified in the validation samples (Figure S7, Supporting Information).

The identification of driver mutations among thousands of passenger mutations is a challenge for applying high‐throughput sequencing as a diagnostic tool for therapeutic decisions.^[^ ^48^ ^]^ The lack of efficient methods for functional screening and validation of observed mutations makes it difficult to confirm the relationship between the identified somatic mutations and clinical response (Figure S8, Supporting Information).^[^ ^49^ ^]^ We, therefore, constructed a custom loss‐of‐function CRISPR/Cas9 library via WES based on deleterious mutations that discriminate responders from non‐responders. We searched for candidates that functionally contribute to drug resistance. Of the 286 library‐enriched genes whose deleterious mutations resulted in resistance to NACT, 15 core genes were retained by generalized linear regressions and binominal logistic model in the final C‐classifier. Most genes were successfully validated via siRNA knockdown assay to confirm the relationship between gene functions and drug resistance. In the validation study, the higher predictive accuracy of the C‐classifier compared with the W‐ and D‐classifiers (AUC, 0.80 versus 0.65 and 0.49, respectively) suggested that integrating the biological experimental data of candidate genes with the generalized linear regressions and binominal logistic model could significantly improve predictive performance (Table 1). This strategy could facilitate more effective application of NACT via patient stratification, representing a valuable progression in the precise management of patients (stage IB to IIB).

Our study identified classifier gene mutations that contribute to chemotherapy resistance in cervical cancer patients. Among these classifier genes, ST6GAL2 is a member of the *β*‐Galactoside *α*2, 6‐sialyltransferase gene (ST6GAL) family. ST6GAL2 has been demonstrated to be a proapoptotic gene, and was significantly hypermethylated in CIN3+ lesions to promote cervical cancer progression.^[^ ^50^ ^]^ POM121, encoding a transmembrane nucleoporin, has been considered to play a key contributor in prostate cancer aggressiveness.^[^ ^51^ ^]^ Commonly mutated POM121 had been found in Mucosal Melanoma,^[^ ^52^ ^]^ and was related to worse clinical outcomes. Other genes such as VPS13C, ANKDD1B, and MID2 have been rarely investigated in drug resistance‐related cancer research. Further investigations of the detailed molecular mechanisms of these 15 core genes could provide new insights into cervical carcinoma drug resistance.

With rapidly advancing technologies and decreasing cost, the 15‐gene panel could be developed into a diagnostic kit that would allow drug response stratification at the time of diagnosis. For NACT‐resistant patients, alternative therapeutic strategies should be provided to avoid unnecessary side effects and delaying effective treatment.^[^ ^53^ ^]^ For NACT‐sensitive patients, induction therapy could reduce tumor size and node positivity and facilitate subsequent local therapy, whether by CCRT or RH.^[^ ^54^ ^]^ The reduction of tumor size would downstage inoperable cancers into resectable ones and make the surgical resection of parametria easier, thus avoiding many surgical complications.^[^ ^55^ ^]^ Another advantage of downstaging is that it could offer a solution for young or pregnant women who wish to preserve their fertility.^[^ ^56^ ^]^ In addition, NACT could decrease the hypoxic cell fraction and increase sensitivity to subsequent CCRT.^[^ ^53^ ^]^ Our panel offers the appropriate selection criteria to include patients who could derive the greatest benefits from NACT and permits patients who are unlikely to benefit from NACT to be quickly transitioned to alternative therapies. Further, our panel may also have the potential prognostic value for the LACC patients. For instance, low ST6GAL2 and VPS13C mRNA expression was found to be associated with poorer prognosis in the TCGA dataset (Figures 5G and 6D), indicating the classifier may also have the ability to predict the prognostic outcome of the patients.

## 4 The Limitation of this Study

A primary limitation of this study was our relatively small sample size of 158 patients in the development and validation set. Additional validation of the classifier using larger samples is therefore required. Second, the predictive role of the multigene classifier on the long‐term survival of LACC patients undergoing NACT remains unknown and warrants further validation in prospective studies and multicenter clinical trials.

## 5 Conclusions

In conclusion, the investigation showed a predictive 15‐gene patient classifier that could accurately identify patients (stage IB to IIB) who would benefit from NACT. The multigene panel developed here is a robust clinical predictor that has the potential to allow for the proper selection of patients for NACT, implicating a significant shift toward individualized therapy.

## 6 Experimental Section

##### Study Design

As shown in Figure S9, Supporting Information, the study consisted of a two‐step approach (discovery and validation). This included exploring candidate genes associated with clinical response to NACT in the discovery cohort, selecting genes compatible with a clinical multigene panel to develop the relevant patient classifier and stratify patients that underwent NACT, and assessing the clinical validity of the multigene panel in specimens from the external cohort.

A total of 56 patients in the NACT group of the GM2010‐06‐02 trial (NCT01267851) were included in the training cohort. Patient‐specific somatic mutations, which led to resistance to NACT, were identified by integrated analysis of WES and CRISPR library screening. For model construction, three drug‐resistant prediction models based on somatic mutations were developed. All 744 candidate genes were used to develop the first model, designated the W‐model. Cancer‐related genes from the CGC were used to build a second model, the D‐model. Finally, markers were reduced to 286 through CRISPR/Cas9 screens, and another prediction model was built and named the C‐model. The classifiers were further validated in the validation cohort of 102 patients with stage IB to IIB from the 2016‐NACT‐01 trial. The clinical response to NACT was determined by WHO criteria after two cycles of treatment. All patients were followed up for 6 weeks (2 cycles). The endpoint of this study was the changes in tumor volume of the patient after receiving 2 cycles of cisplatin‐based NACT.

##### Patients

The investigation was conducted between 1st January 2016 and 1st February 2019. For the retrospective discovery study, patients were identified from the Clinical Database and Biobank^[^ ^32^ ^]^ (ClinicalTrials.gov numbers, NCT01267851) based on a 1:1 (case: control) ratio. The case referred to the non‐responder, and the control referred to the responder in the study. For the prospective validation cohort, patients with FIGO stage IB to IIB cervical cancer were recruited at either Tongji Hospital or the Central Hospital of Wuhan in China (ClinicalTrials.gov numbers, NCT03229187). Patients received two cycles of paclitaxel (165–175 mg/m^2^) and cisplatin (75–80 mg/m^2^) once every 3 weeks. Dynamic change in tumor size was evaluated by vaginal ultrasound during each cycle of treatment and randomly verified with MRI. All patients gave informed consent for molecular and genetic analysis, and the study was approved by the local regional ethics committee. Samples were collected from the primary tumor prior to NACT. Patients were excluded on predefined criteria: lack of tumor‐blood pairs and insufficient quantity and/or quality of DNA.

In this study, the focus was on squamous cell carcinomas because they represented a major pathological type of cervical cancer. Second, due to the sample volume of adenocarcinomas was small (only 8 cases in the validation cohort study), it was relatively hard to achieve statistic power to draw any conclusions. Considering the above reasons, patients with squamous cell carcinomas were included in this investigation.

##### Sample Size

Based on previous related studies,^[^ ^57^, ^58^ ^]^ it was assumed that prediction model AUC would reach 0.7, with a null hypothesis of 0.5, sample allocation ratio of 4, a power of 0.8, and type I error set at 5%. 100 Patients were needed in the validation cohort. The validation cohort finally included 102 patients.

##### Sample Preparation

Formalin‐fixed, paraffin‐embedded (FFPE) tissue blocks were cut into 3 µm for H&E staining and examined by a pathologist to select tumor samples with malignant cell purities of over 70%. Then, for each FFPE sample, 6 slices of 10 µm were cut and placed in a 1.5 mL Eppendorf tube, according to the manufacturer's protocol. DNA from tumor was extracted using commercial kits (GeneRead DNA FFPE kit, Qiagen), and DNA from match blood was extracted using a standard Qiagen DNA extraction kit. DNA was quantified using a Qubit 2.0 Fluorometer (Life Technologies) (total DNA > 2 µg), NanoDrop One/One^C^ (1.8 < 260/280 < 2.0). Fragment length and degradation were assessed using Agilent High Sensitivity DNA Kit (Agilent 2100 Bioanalyzer) (average fragment size > 500bp). DNA was stored at −20 °C.

##### Genome‐Wide Copy Number Analysis

Hybridization buffers were prepared and array hybridization was performed in a hybridization over (Cat#00‐0331‐220V, Affymetrix, Santa Clara, CA, US). After 16 h hybridization, arrays were washed in a Fluidics Station (Cat#00‐0079, Affymetrix, Santa Clara, CA, US) according to the Affymetrix OncoScan Assay User Manual (Cat#703 038 Rev. 3, Affymetrix, Santa Clara, CA, US). Arrays were scanned with a GeneChip Scanner 3000 (Cat#00‐00212, Affymetrix, Santa Clara, CA, US) and Command Console Software 3.1 (Affymetrix, Santa Clara, CA, US) with default settings. Raw data passed quality control were further analyzed by Affymetrix OncoScan Analysis Suite (Affymetrix, Santa Clara, CA, US). Affymetrix single‐nucleotide polymorphism arrays were analyzed to call CNV using Chromosome Analysis Suite software (ChAs, v3.3.0), with default parameters. For each sample, “germline” CNV was called if it was observed in the blood sample, “somatic” CNV was called if the event was not observed in the corresponding normal (blood) sample. Data were filtered referring to the following conditions: gain or loss of CNV fragments > 50 kb with at least 25 consecutive probes contained in the window.

##### Whole‐Exome Sequencing (WES)

Capture libraries were prepared from 2 µg genomic DNA (gDNA) using the Agilent SureSelect Human All Exon V6 kit (Agilent Technologies) following the manufacturer's recommendations. Fragmentation was carried out by a hydrodynamic shearing system (Covaris, Woburn, MA) to generate 180–280 bp fragments. DNA fragments with ligated adapter molecules on both ends were selectively enriched using PCR followed by liquid‐phase hybridization using biotin‐labeled probes. A total of 60 Mb sequences of the whole human exome were captured. Libraries were quantified and sequenced on the Illumina HiseqX platform for a mean coverage of 200×.

##### Identify Candidate Genes Related to Drug Response in Training Cohort

A retrospective cohort of 30 non‐responders and 26 responders was used to find out somatic mutations resistant to NACT treatment. Whole‐exome sequencing (WES), which covers most of the human coding genes, was applied for. Sequence reads were analyzed according to GATK best practices.^[^ ^59^ ^]^ Paired‐end reads were mapped to the reference genome (UCSC hg19) with BWA‐MEM (v0.7.8).^[^ ^60^ ^]^ Picard tools were employed to mark PCR duplicate reads, and the Indel Realigner algorithm (GATK v3.8.0) was used to improve alignment accuracy. The MuTect2 (GATK v3.8.0)^[^ ^61^ ^]^ was used to detect somatic mutations in tumor‐control paired samples. High confidence variants were annotated with ANNOVAR (v2015Mar22).^[^ ^62^ ^]^ To remove any possible germline contamination, somatic SNVs and indels with population frequency greater than 0.01 in 1000G/EXAC/ESP6500^[^ ^47^ ^]^ were filtered. Associations between non‐silent somatic mutations (missense mutations, nonsense mutations, nonstop mutations, frame‐shift/in‐frame insertions or deletions (indels), and splice‐site mutations) and NACT responses were evaluated by Fisher's exact test. Enrichment analyses were then performed to filter genes that had deleterious mutations in at least two non‐responders, and identified 744 genes with Mut_insen–Mut_sen ≥ 2 and *p*‐value < 0.25.

##### Gene Set Enrichment Analysis

Using the function gometh from the R‐package clusterprofile,^[^ ^63^ ^]^ gene set enrichment analysis of the 744 genes screened based on WES was performed. Multiple testing correction was applied using the BH method (false discovery rate, FDR < 5%).

##### Screening through CRISPR/Cas9 Library

The CRISPR/Cas9 library screening was conducted as described in nature protocols.^[^ ^64^ ^]^ Lentiviral products were obtained by co‐transfection of library plasmids with three viral packaging plasmids into HEK293T using the polyethylenimine (PEI) method. HEK293T cells were grown in 10 cm dishes to 40% confluence. For each dish, transfection was performed using 28 µL of PEI (Promega), 4.2 µg of pLP1, 2.1 µg of pLP2, 3.1 µg of pVSVG, and 4 µg of library plasmids. After 30 min of incubation at room temperature, the mixture was added to the cells. After 48 h of infection, harvested viruses were passed through a 0.45 µm filter. To identify genes whose inhibition desensitized cells to treatment, screens were performed in a panel of SiHa cells, which were derived from a human papilloma virus‐induced cervical squamous carcinoma. The SiHa cell line was seeded at 400 000 cells per well in six‐well plates and the next day transduced at a multiplicity of infection (MOI) of 0.2. Transduced cells were then maintained at 300x coverage of the library in puromycin selection for 3 days to allow for the generation of knockout cells. Then cells were split into control and drug treatment conditions in the dose of drugs: DDP (10 µm), DDP (10 µm) plus Taxol (30 nm), and Taxol (30 nm), respectively, each with three replicates. Fresh DMEM with toxins were changed every 3 days. After 2 weeks, cells were collected separately for gDNA extraction, followed by PCR amplification of the sgRNA‐coding region and deep‐sequencing analysis.

sgRNA tags were aligned to human reference (hg19) using BWA (v0.7.8), and read counts for 724 target genes were estimated by bedtools (v2.19.1).^[^ ^65^ ^]^ The R software package from Bioconductor, DESeq2,^[^ ^66^ ^]^ was used to perform the differential analysis of CRISPR/Cas9 screens treated with DDP, Taxol, and combination with DDP and Taxol. sgRNA enrichment selected for further analysis was based on the below criteria: i) sgRNAs with fold changes > 1 and *p*‐values < 0.1 for both two independent replications in DDP and Taxol groups, or ii) sgRNAs with fold change > 1.32 and *p*‐values < 0.1 for either two independent replications in Taxol and combination with DDP group. (CRISPR‐Cas9 Screening and data analysis was completed by Generulor Company Bio‐x Lab, Guangzhou 510 006, Guangdong, China)

##### Constructing a Genomic Classifier by Generalized Linear Regressions and Binominal Logistic Model

Shrinkage methods including Least absolute shrinkage and selection operator (LASSO),^[^ ^67^ ^]^ Elastic Net, and Ridge Regression are the three main approaches of variable selection with high‐dimensional predictors, especially LASSO. Before model construction, the mutation status of each gene was transformed into 0 (no mutation) or 1 (non‐silent somatic mutation) to consist of observed data for all samples. For C‐classifier, W‐classifier, and D‐classifier, three generalized linear regression were conducted consecutively and multicollinearity was checked by Spearman's correlation after each regression to exclude genes of severe correlation with other variables. First, Ridge regressions were conducted 200 times (tenfold cross‐validation and *λ* with minimum of mean squared errors); the coefficients of non‐zero genes were summed up and according to the distribution, top 80% genes were adopted(n = 222). Second, Elastic Net regression was conducted 200 times (tenfold cross‐validation and *λ* with minimum of mean squared errors); the coefficients of non‐zero genes were summed up and according to the distribution, top 90% genes were adopted(n = 58). Third, Shrinkage methods including LASSO regression were conducted 200 times(tenfold cross‐validation and *λ* with minimum of mean squared errors); the coefficients of non‐zero genes were summed up and according to the distribution, top 75% genes were adopted (n = 32).

Before conducting a final logistic binomial model, two consecutive co‐occurrence checks were conducted and excluded 5 genes that were highly correlated to other variables. Finally, the CRISPR model was built using the 27 genes by logistic binomial model and tenfold cross‐validation strategy. Generally, the top 2 ranked genes were fixed and added other genes one by one. At each step, genes with the highest AUC performances were added, and excluded genes with the same AUC addition powers yet i) lower LASSO coefficient ranks or ii) higher frequencies of positive correlations to other genes (Spearman *r* ≥ 0.5). Gene addition step was stopped when the AUC power no longer increased. A risk score was calculated for each patient using a formula derived from the mutational status of C‐classifier 15 genes weighted by their regression coefficient:

Risk score = −1.9 + (21.8 × Mut‐ST6GAL2) + (21.4×Mut‐POM121) + (21.3 × Mut‐MID2) + (21.1 × Mut‐MIDN) + (20.8 ×Mut‐HDAC11) + (20.7 × Mut‐FOXD4L3) + (20.7 × Mut‐MTMR9) + (20.6 × Mut‐WIPF2) + (20.5 × Mut‐SEMA4C) + (19.0 × Mut‐COL18A1) + (15.9 × Mut‐RLF) + (4.7 ×Mut‐SALL3) + (2.4 × Mut‐ANKDD1B) + (1.6 × Mut‐BCL9L) + (1.3 × Mut‐VPS13C).

Binary matrices for the presence or absence of a gene's mutation were constructed, and glmnet package (R environment 3.3.3) was used to perform LASSO, Elastic Net, and Ridge regression analysis. ROC curve was plotted and used to assess the ability to distinguish between responder and non‐responder. The optimal cut‐off value was chosen as the maximum of Youden's index for training cohort. Then, using the same formula, the original 744 genes were applied to develop W‐classifier. Last, the 744 genes were then narrowed down to cancer driver genes from the CGC to develop D‐classifier. The genomic classifiers were applied to samples from biopsy tissue prior to NACT, without any prior knowledge of the therapeutic response labels for this cohort. The patient was considered a non‐responder with non‐silent somatic mutation in any one of the model genes.

##### Constructing a Clinical Classifier

Binomial logistic regression was used, implemented in the R glm method, to build the predictive model of NAC response bases on four clinical variables: age, pathological differentiation, clinical stage, and SCC‐pretreatment. The sample with missing value was excluded. Age was divided into two categories (old/young) via the median (50 yr). ROC curve was plotted using pROC, and the optimal cut‐point was determined by Youden's index method in cut‐point.

##### siRNA Transfection and Validation Drug Screen

Cells were transfected with Lipofectamine 3000 and siRNA according to the manufacturer's protocol. Every gene was targeted with 3 siRNAs. Knockdown was validated by qRT‐PCR. Validation drug screen in SiHa was conducted with 2 siRNAs/gene. Cell viability was analyzed using the Cell Counting Kit‐8 (Dojindo), according to the manufacturer's instructions. Briefly, after transfected with siRNA, 5 × 10^3^ SiHa cells were plated in triplicate in 96‐well plates and allowed to adhere for 24 h. Then cells were treated with increasing concentrations of cisplatin (0 to 100µm) or Paclitaxel (0 to 1µm) for 48 h. Viability was quantified by reading the absorbance at 450 nm in SpectraMax Microplate reader (Molecular Devices). The data were analyzed using GraphPad Prism 7 software.

##### IHC

IHC was performed on paraffin‐embedded slides. Briefly, deparaffinized slides were rehydrated and incubated with a tris‐EDTA (pH 9.0) (10 mm tris base, 1 mm EDTA, and 0.05% Tween 20) antigen retrieval solution in a Pressure Cooker. Nonspecific antibody binding was blocked with 10% bovine serum albumin (Servicebio) for 1 h, followed by overnight incubation with anti‐VPS13C (1:300; abcam, ab130399), anti‐ST6GAL2 (1:20; R&D Systems, AF7747‐SP) primary antibodies. To visualize antigens, slides were washed and incubated for 1 h with horseradish peroxidase (HRP)–conjugated anti‐rabbit (antgene, ANT020) and DAB chromogen. All images were processed from Adobe Photoshop CC 2017.

##### Statistical Analysis

The number of copy gains/losses present in the non‐response group versus response group in 1 Mbp windows was contrasted along the human genome using a two‐sided Fisher's exact test, implemented with SciPy (v1.1.0) module in Python (v2.7). Fisher's exact test was applied to analyze associations between somatic events and drug response and to calculate the odds ratio of drug resistance for each gene. PCA was performed with the prcomp function in the R environment. The hierarchical clustering method was used to cluster discovery samples based on data of CNV or somatic mutations and was visualized with the R package pheatmap. In CRISPR screening, the enrichment of sgRNA was ranked by the average fold‐change of normalized counts: reads^Exp^/reads^Ctrl^. The adjusted *p*‐value was calculated to evaluate the data quality. The pROC package was used to calculate the significance of differences in the AUC. Standard logistic regression was used to estimate the odds ratio and 95% CI for evaluating the performance of the predictive model. Significance was based on *p* < 0.05 and 95% CI estimates.

## Conflict of Interest

The authors declare no conflict of interest.

## Supporting information

Supporting Information

Supporting Table 4

Supporting Table 5

Supporting Table 6

Supporting Table 5

Supporting Table 8

Supporting Table 9

Supporting Table 8

## Data Availability

The data that support the findings of this study are available from the corresponding author upon reasonable request.
