# Supplementary material for: A Fifteen‐Gene Classifier to Predict Neoadjuvant Chemotherapy Responses in Patients with Stage IB to IIB Squamous Cervical Cancer
Source: Adv Sci (Weinh). 2021 Mar 18;8(10):2001978. doi: 10.1002/advs.202001978 (PMC8132153; doi:10.1002/advs.202001978)
Supplement: Supplementary file 1 — Supporting Information [file ADVS-8-2001978-s002.pdf]

© 2021 Wiley-VCH GmbH

ADVANCED SCIENCE
Open Access

Supporting Information

for *Adv. Sci.*, DOI: 10.1002/advs.202001978

A Fifteen-Gene Classifier to Predict Neoadjuvant Chemotherapy Responses in Patients with Stage IB to IIB Squamous Cervical Cancer

Xun Tian[1,2†], *Xin Wang*[1,†], *Zifeng Cui*[3,†], *Jia Liu*[1,†], *Xiaoyuan Huang*[1,†], *Caixia Shi*[5,†], *Min Zhang*[6,†], *Ting Liu*[2], *Xiaofang Du*[2], *Rui Li*[2], *Lei Huang*[2], *Danni Gong*[2], *Rui Tian*[3], *Chen Cao*[2], *Ping Jin*[2], *Zhen Zeng*[2], *Guangxin Pan*[2], *Meng Xia*[2], *Hongfeng Zhang*[4], *Bo Luo*[4], *Yonghui Xie*[4], *Xiaoming Li*[1], *Tianye Li*[1], *Jun Wu*[6], *Qinghua Zhang*[2,*], *Gang Chen*[1,*], *Zheng Hu*[1,2,3,*]

Supporting Information

A Fifteen-Gene Classifier to Predict Neoadjuvant Chemotherapy Responses in Patients with Stage IB to IIB Squamous Cervical Cancer

The PDF file includes:

Figure S1. Response Outcome with WHO criteria and RECIST criteria. ---------- 3
Figure S2. Copy number variations in cervical cancer. ---------- 4
Figure S3. Gene Ontology (GO) terms significantly enriched. ---------- 5
Figure S4. Gene knockdown effects on response to cisplatin/paclitaxel. ---------- 6
Figure S5. The AUC and accuracy performances of 500 times down-sample from 102 validation samples. ---------- 7
Figure S6. Risk score by the classifiers in the validation set. ---------- 8
Figure S7. 744 mutation genes validated in the validation samples. ---------- 9
Figure S8. Somatic mutation analysis in the training cohort and the validation cohort. ---------- 10
Figure S9. Study flowchart. ---------- 11
Table S1. Patient characteristics of neoadjuvant chemotherapy training cohort. ---------- 12
Table S2. Clinical response ratio of neoadjuvant chemotherapy group. ---------- 13
Table S3. Patient characteristics of neoadjuvant chemotherapy validation cohort. ---------- 14
Table S11. REMARK List. ---------- 15

Other Supplementary Material for this manuscript includes the following:

Data file S1 contains the following supplementary tables:

Table S4 (Microsoft Excel format). List of significant copy number variations (deletions and amplifications), deletion-amplification frequency and somatic mutations and frequency in training samples.

Table S5 (Microsoft Excel format). Correlation between copy number variation of cisplatin/paclitaxel resistant gene or driver genes and the response to neoadjuvant chemotherapy (NACT) in cervical cancer.

Table S6 (Microsoft Excel format). List of somatic mutations selected by Hierarchical Clustering in training samples.

Table S7 (Microsoft Excel format). List of somatic mutations were selected for candidate genes.

Table S8 (Microsoft Excel format). GO analysis in the training cohort.

Table S9 (Microsoft Excel format). List of somatic mutations screening through CRISPR library in cisplatin/paclitaxel.

Table S10 (Microsoft Excel format). Algorithm 15 genes of C-classifier.

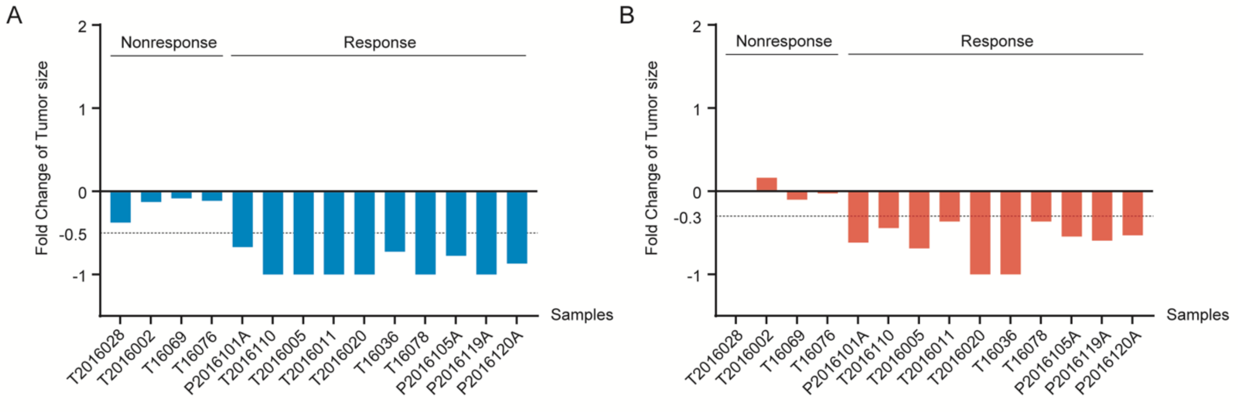

Figure S1. Response Outcome with WHO criteria and RECIST criteria.

(A) Response outcome with WHO criteria. A-0.5-fold change of tumor was the demarcation line of chemotherapy response based on WHO criteria. (B) Response outcome with RECIST criteria. A-0.3-fold change of tumor was the demarcation line of chemotherapy response based on RECIST criteria.

Y > 0 represents tumor size increase, and Y < 0 represents tumor size decrease. Fold-change of tumor size was calculated by the ratio of tumor size after and tumor before chemotherapy.

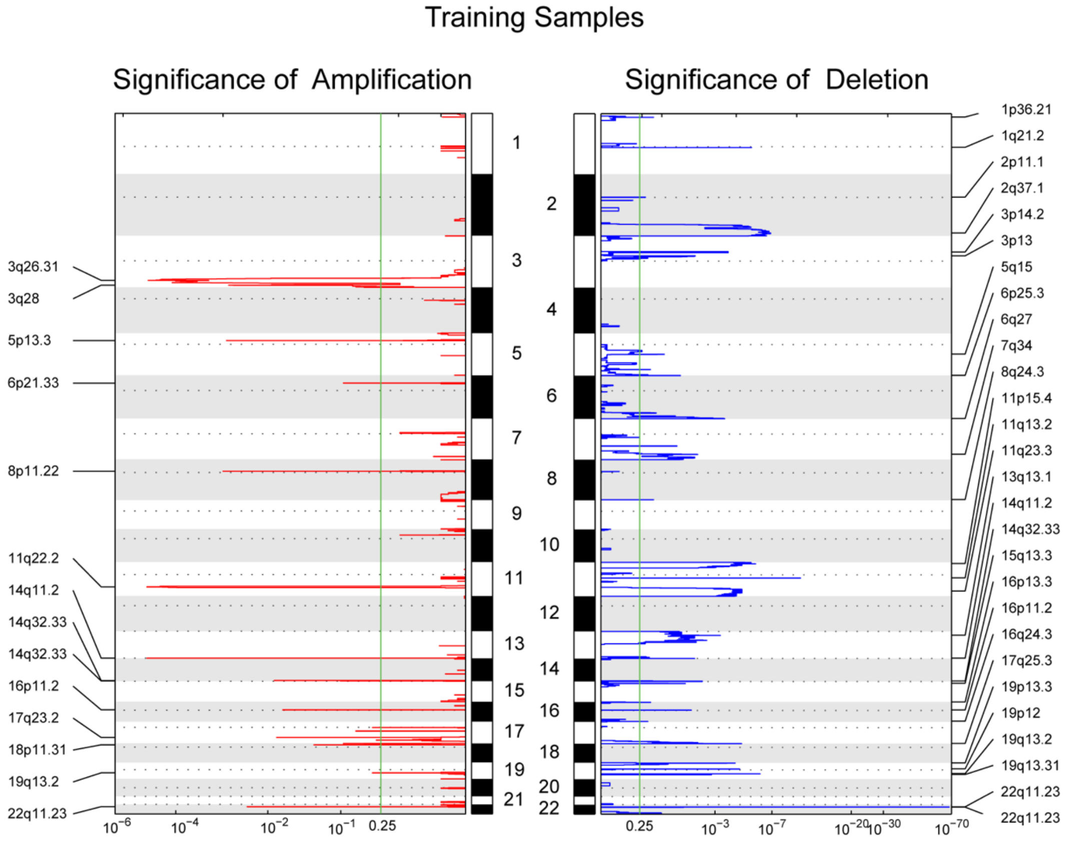

Figure S2. Copy number variations in cervical cancer.

Chromosomal locations for peaks of significantly recurrent focal amplifications (red) and deletions (blue) are plotted by −log10 q value for all core set samples. Peaks are annotated with cytoband (Fisher's exact test with a false discovery rate threshold of 0.25).

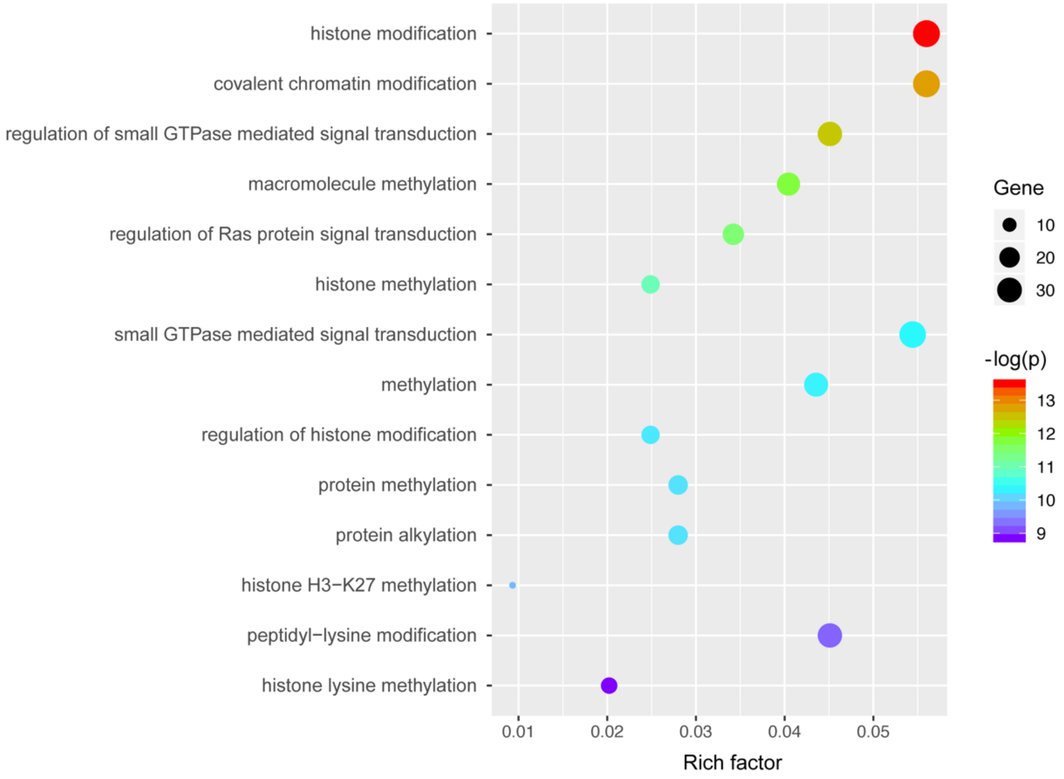

Figure S3. Gene Ontology (GO) terms significantly enriched.

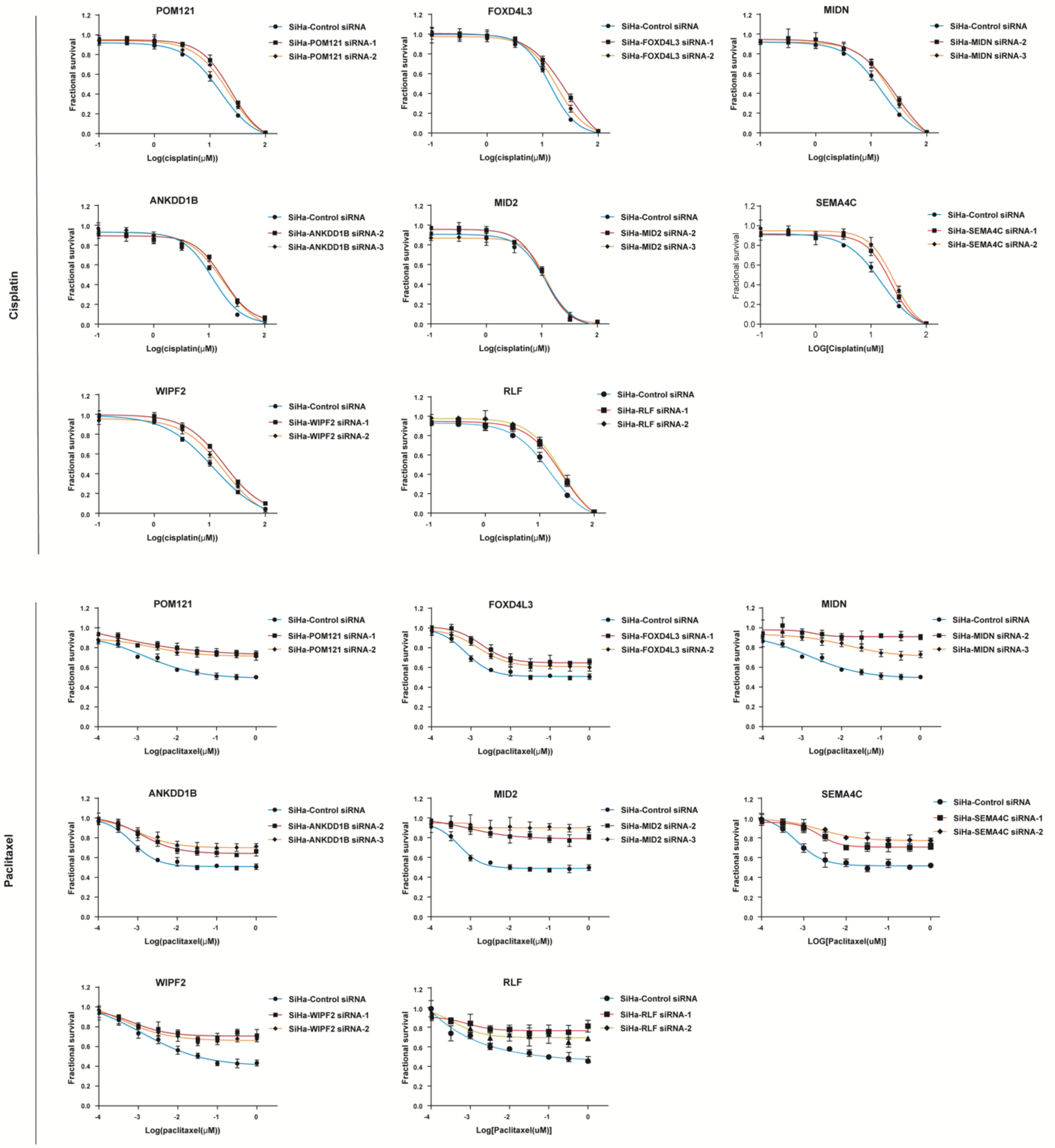

Figure S4. Gene knockdown effects on response to cisplatin/paclitaxel.

Cell viability was determined after SiHa cell transfection with siRNA. After 24 h, cells were reseeded for Cisplatin/Paclitaxel sensitivity assays. CCK-8 was used to determine the percentage of cell viability after 48 h of Cisplatin/Paclitaxel treatment. The X axis corresponds to the Log value of Cisplatin/Paclitaxel (μM) and the Y axis to the percentage of viable cell.

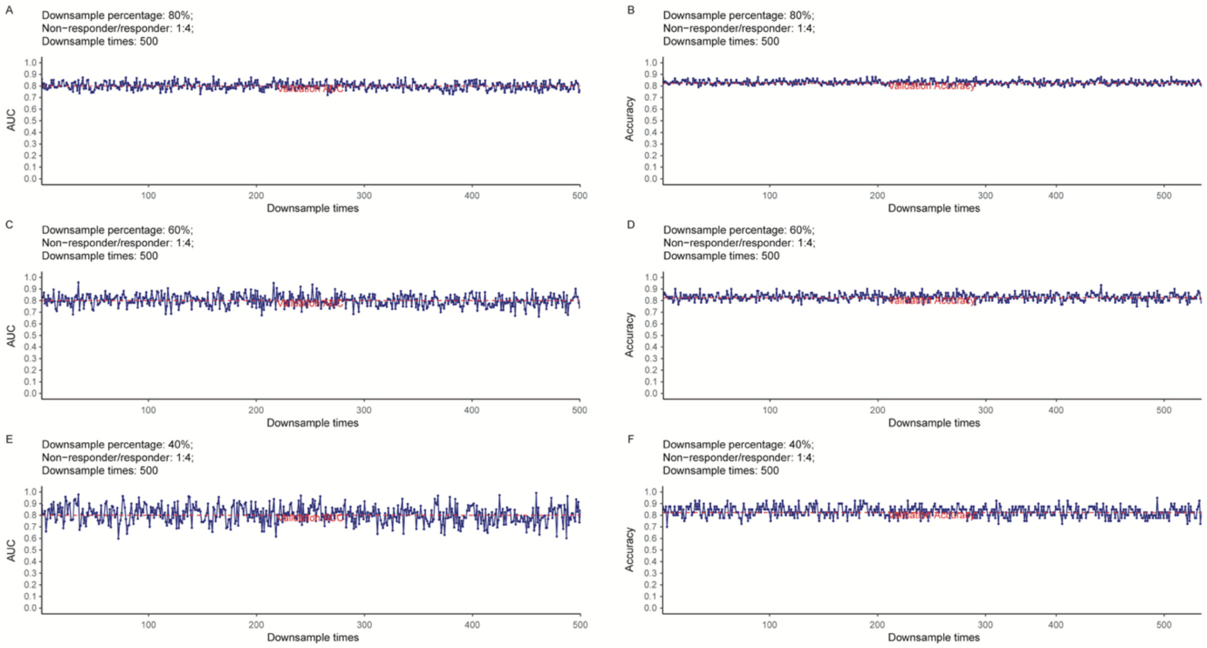

Figure S5. The AUC and accuracy performances of 500 times down-sample from 102 validation samples.

A, C, E. The AUC performances of 500 times down-sample from 102 validation samples with the percentage of 40% (A), 60% (C) and 80% (E).

B, D, F. The accuracy performances of 500 times down-sample from 102 validation samples with the percentage of 40% (B), 60% (D) and 80% (F).

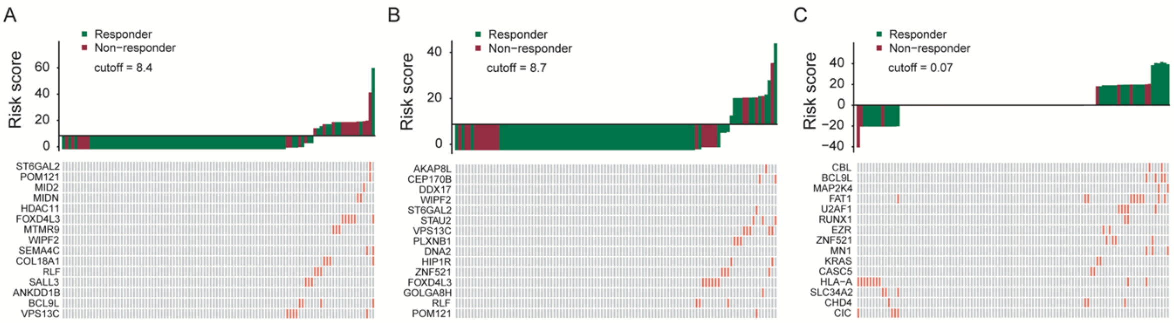

Figure S6. Risk score by the classifiers in the validation set.

A. C-classifier, the patients were divided into non-responder and responder groups using the cut-off value of the classifier risk score (8.4). B. W-classifier, the patients were divided into non-responder and responder groups using the cut-off value of the classifier risk score (8.7). C. D-classifier, the patients were divided into non-responder and responder groups using the cut-off value of the classifier risk score (0.07). Upper-left panel: risk score distribution of the 15-gene-based classifier and the response status of 102 patients. Lower-left panel: the status of the fifteen genes in the 102 patients of the validation set.

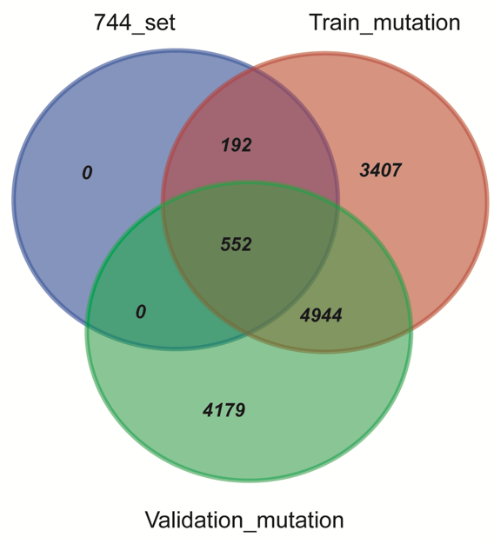

Figure S7. 744 mutation genes validated in the validation samples.

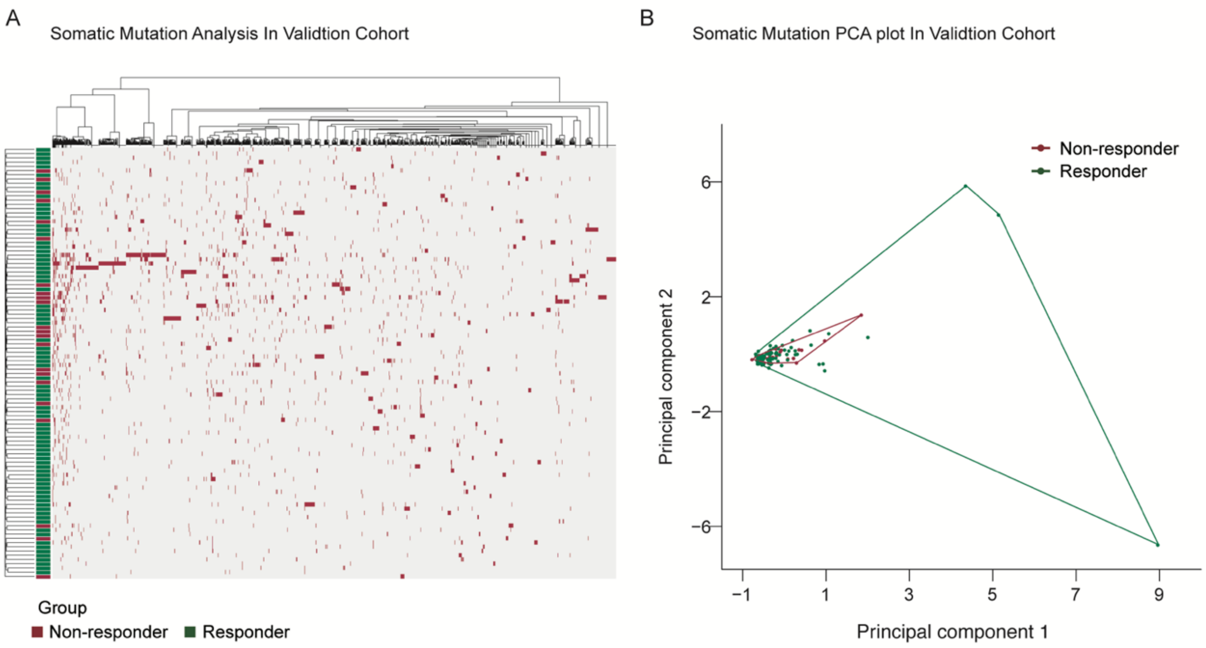

Figure S8. Somatic mutation analysis in the training cohort and the validation cohort.

(A) Heatmaps of unsupervised hierarchical clustering of somatic mutations in the validation cohort. Bar colors indicate clinical response to neoadjuvant chemotherapy (NACT): red indicates a non-responder, and green indicates a responder. Colored rectangles on heatmaps show single nucleotide variations (SNVs)/Indels. (B) Principal component analysis (PCA) of somatic mutations in the validation cohort.

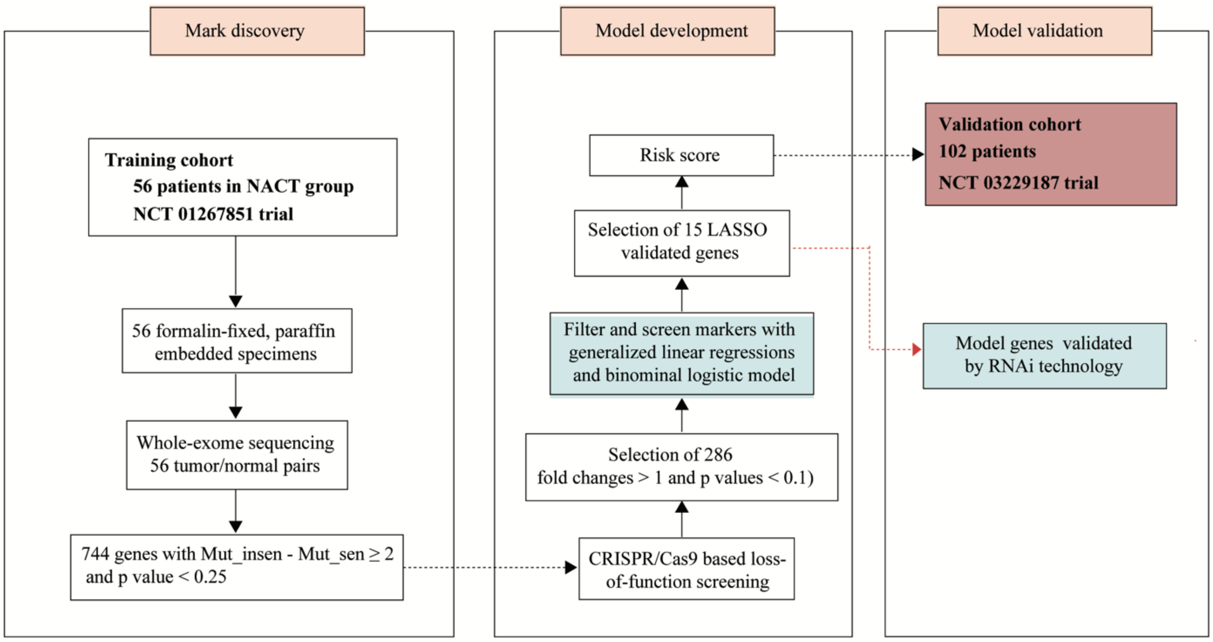

Figure S9. Study flowchart.

Table S1. Patient characteristics of neoadjuvant chemotherapy training cohort.

Parameters	Non-Responders (n = 30)	Responders (n = 26)	*P*
Median Age-yr (IQR)	50 (45.75-54)	48 (44.25-56.5)	.9921[A]
Differentiation, No. (%)			.5099[B]
Well	2 (6.67)	0 (0)	
Moderate	13 (43.33)	12 (46.15)	
Poor	10 (33.33)	11 (42.31)	
Unknown	5 (16.67)	3 (11.54)	
FIGO Stage, No. (%)			.4756[B]
IB	10 (33.33)	9 (34.62)	
IIA	5 (16.67)	6 (23.08)	
IIB	15 (50.00)	11 (42.31)	
Mean Tumor diameter-cm	4.4	4.6	.6391[A]
Lymph node metastasis, No. (%)			.0005[B*]
Negative	13 (43.33)	24 (92.31)	
Positive	12 (40.00)	2 (7.69)	
Unknown	5 (16.67)	0 (0)	
Mean SCC-μg/L	6.0	3.0	.2705[A]

Note: A Student's t test; B Chi-square test; *$P < 0.05$; Percentages may not total 100, because of rounding. Abbreviations: interquartile range, IQR.

Table S2. Clinical response ratio of neoadjuvant chemotherapy group.

	Training cohort (n = 56)					Validation cohort (n = 102)				
	CR+PR	CR	PR	SD	PD	CR+PR	CR	PR	SD	PD
No. (%)	26 (46.43)	1 (1.79)	25 (44.64)	29 (51.79)	1 (1.79)	80 (78.43)	34 (33.33)	46 (45.10)	18 (17.65)	4 (3.92)
FIGO stage, No. (%)										
IB	9 (16.07)	0 (0)	9 (16.07)	10 (17.86)	0 (0)	16 (15.69)	4 (3.92)	12 (11.76)	2 (1.96)	1 (0.98)
IIA	6 (10.71)	0 (0)	6 (10.71)	5 (8.93)	0 (0)	21 (20.59)	12 (11.76)	9 (8.82)	8 (7.84)	1 (0.98)
IIB	11 (19.64)	1 (1.79)	10 (17.86)	14 (25.00)	1 (1.79)	43 (42.16)	18 (17.65)	25 (24.51)	8 (7.84)	2 (1.96)

Abbreviations: complete response, CR; partial response, PR; stable disease, SD; progressive disease, PD.
Note: Percentages may not total 100, because of rounding.

Table S3. Patient characteristics of neoadjuvant chemotherapy validation cohort.

	**NACT validation cohort**
Patients, No.	102
Median Age-yr (IQR)	52 (46-55)
Differentiation, No. (%)	
Moderate	43 (42)
Poor	41 (40)
Unknown	18(18)
FIGO Stage, No. (%)	
IB	19 (19)
IIA	30 (29)
IIB	53 (52)
Mean Tumor diameter-cm	4.0
Complete pathological response, No. (%)	15 (15)
Lymph node metastasis, No. (%)	
Negative	82 (80)
Positive	18 (18)
Unknown	2 (2)
Mean SCC-μg/L	10.3

Note: Percentages may not total 100, because of rounding.
Abbreviations: interquartile range, IQR.

Table S11. REMARK List.

	Item to be reported	Page no.
**INTRODUCTION**		
1	State the marker examined, the study objectives, and any pre-specified hypotheses.	5 (Line 102-107)
**MATERIALS AND METHODS**		
Patients		
2	Describe the characteristics (e.g., disease stage or co-morbidities) of the study patients, including their source and inclusion and exclusion criteria.	21-22 (Line 447-466), Figure 1, Table S1, Table S3
3	Describe treatments received and how chosen (e.g., randomized or rule-based).	21-22 (Line 453-455,458)
Specimen characteristics		
4	Describe type of biological material used (including control samples) and methods of preservation and storage.	22-23 (Line 473-482)
Assay methods		
5	Specify the assay method used and provide (or reference) a detailed protocol, including specific reagents or kits used, quality control procedures, reproducibility assessments, quantitation methods, and scoring and reporting protocols. Specify whether and how assays were performed blinded to the study endpoint.	24 (Line 500-507) 25-26 (Line 530-558) 28 (Line 602-603)
Study design		
6	State the method of case selection, including whether prospective or retrospective and whether stratification or matching (e.g., by stage of disease or age) was used. Specify the time period from which cases were taken, the end of the follow-up period, and the median follow-up time.	21 (Line 443-444) 21 (Line 447-453)
7	Precisely define all clinical endpoints examined.	21 (Line 444-445)
8	List all candidate variables initially examined or considered for inclusion in models.	21 (Line 439) Table S9 29 (Line 608-609)
9	Give rationale for sample size; if the study was designed to detect a specified effect size, give the target power and effect size.	22 (Line 468-471)
Statistical analysis methods		
10	Specify all statistical methods, including details of any variable selection procedures and other model-building issues, how model assumptions were verified, and how missing data were handled.	27-29 (Line 563-612)
11	Clarify how marker values were handled in the analyses; if relevant, describe methods used for cut-point determination.	25-29 (Line 520-521, 587-598, 608-612)

**RESULTS**		
Data		
12	Describe the flow of patients through the study, including the number of patients included in each stage of the analysis (a diagram may be helpful) and reasons for dropout. Specifically, both overall and for each subgroup extensively examined report the numbers of patients and the number of events.	Figure 1
13	Report distributions of basic demographic characteristics (at least age and sex), standard (disease-specific) prognostic variables, and tumor marker, including numbers of missing values.	Table S1, Table S3
Analysis and presentation		
14	Show the relation of the marker to standard prognostic variables.	Figure 4, 7
15	Present univariable analyses showing the relation between the marker and outcome, with the estimated effect (e.g., hazard ratio and survival probability). Preferably provide similar analyses for all other variables being analyzed. For the effect of a tumor marker on a time-to-event outcome, a Kaplan-Meier plot is recommended.	16<br>(Line 341-345)<br>Figure 7E
16	For key multivariable analyses, report estimated effects (e.g., hazard ratio) with confidence intervals for the marker and, at least for the final model, all other variables in the model.	17<br>(Line 345-348)<br>Figure 7E
17	Among reported results, provide estimated effects with confidence intervals from an analysis in which the marker and standard prognostic variables are included, regardless of their statistical significance.	Table 1(Page 37)
18	If done, report results of further investigations, such as checking assumptions, sensitivity analyses, and internal validation.	16<br>(Line 334-337)<br>27<br>(Line 565-578)<br>Table 1(Page 37)
**DISCUSSION**		
19	Interpret the results in the context of the pre-specified hypotheses and other relevant studies; include a discussion of limitations of the study.	17-20<br>(Line 354-418)
20	Discuss implications for future research and clinical value.	19<br>(Line 394-407)
